# Astragalin from *Thesium chinense*: A Novel Anti-Aging and Antioxidant Agent Targeting IGFR/CD38/*Sirtuins*

**DOI:** 10.3390/antiox13070859

**Published:** 2024-07-18

**Authors:** Ruifeng Wang, Anping Ding, Jiaye Wang, Jiaxue Wang, Yujie Zhou, Miao Chen, Shuang Ju, Mingpu Tan, Zengxu Xiang

**Affiliations:** 1 College of Horticulture, Nanjing Agricultural University, Nanjing 210095, China; 2021804292@stu.njau.edu.cn (R.W.); 2021804301@stu.njau.edu.cn (A.D.); 2023804373@stu.njau.edu.cn (Y.Z.); 2023104142@stu.njau.edu.cn (M.C.); 2023804372@stu.njau.edu.cn (S.J.); 2 College of Pharmacy, Nanjing Medical University, Nanjing 211166, China; wangjiaye@stu.njmu.edu.cn; 3 College of Life Sciences, Nanjing Agricultural University, Nanjing 210095, China; 2023816123@stu.njau.edu.cn

**Keywords:** astragalin, zebrafish embryos, oxidative stress, *C. elegans*, antioxidant

## Abstract

Astragalin (AG), a typical flavonoid found in *Thesium chinense* Turcz (*T. chinense*), is abundant in various edible plants and possesses high nutritional value, as well as antioxidant and antibacterial effects. In this study, we initially predicted the mechanism of action of AG with two anti-aging and antioxidant-related protein targets (CD38 and IGFR) by molecular docking and molecular dynamics simulation techniques. Subsequently, we examined the anti-aging effects of AG in *Caenorhabditis elegans* (*C. elegans*), the antioxidant effects in zebrafish, and verified the related molecular mechanisms. In *C. elegans*, AG synergistically extended the lifespan of *C. elegans* by up-regulating the expression of *daf-16* through inhibiting the expression of *daf-2/IGFR* and also activating the AMPK and MAPK pathways to up-regulate the expression of *sir-2.1*, *sir-2.4*, and *skn-1*. In oxidatively damaged zebrafish embryos, AG demonstrated a synergistic effect in augmenting the resistance of zebrafish embryos to oxidative stress by up-regulating the expression levels of *SIRT1* and *SIRT6* within the zebrafish embryos system via the suppression of CD38 enzymatic activity and then inhibiting the expression of *IGFR* through high levels of *SIRT6*. These findings highlight the antioxidant and anti-aging properties of AG and indicate its potential application as a supplementary ingredient in aquaculture for enhancing fish health and growth.

## 1. Introduction

*T. chinense*, a small perennial and hemi-parasitic plant of the family *Santalaceae*, is distributed in Africa, Europe, Asia, and America [1]. Thirty-four chemical constituents, including flavonoids, alkaloids, and terpenoids, were identified from *T. chinense*. Among them, flavonoids were the main and characteristic components. Modern pharmacological studies have proven that *T. chinense* offers excellent antioxidant [2], anti-inflammatory [3], and antibacterial [4] properties. AG, found in a variety of vegetables and fruits (*Malus halliana* [5], *African Cabbage* [6], *Rosa canina*, *Rosa sempervivens*, *Pyrocantha coccinea* [7], and fruits of *Lycium barbarum* [8]), is a typical flavonoid from *T. chinense* and possesses Estrogen-increasing [9], procoagulant [10], and anti-neuroinflammatory effects [11].

*C. elegans* has been extensively utilized globally as a preferred model organism for the evaluation of bioactive compounds that exhibit effects on longevity [12,13] and has revealed numerous breakthrough discoveries in the field of antioxidant research [14].

In addition, zebrafish embryos, a convenient, reliable, and inexpensive animal model for statistically dose-dependent toxicological studies [15], have been commonly used to screen plant bioactive constituents for antioxidant activity [16]. Based on the high conservation of *daf-2*/*IGFR* between *C. elegans* and mammals [17], researchers found that biologically active compounds with inhibitory effects on *IGFR* could reduce *IIS* signaling in *C. elegans* and thus up-regulate the transcription factor *daf-16*/FOXO [18]. For example, *Oleuropein* enhances stress resistance and extends lifespan by inhibiting the expression of *daf-2*/*IGFR* in *C. elegans* [19].

The concept of hormesis is of great importance in the application of nutritional antioxidants. Hormesis action refers to a biphasic dose response in which a low dose of a harmful substance or stress can actually induce an adaptive response in an organism that enhances its resilience and self-protection. For nutritional antioxidants, this means that small doses of antioxidant components can activate endogenous cellular defense mechanisms, such as up-regulating the expression of antioxidant enzymes and repair enzymes, thereby enhancing the antioxidant and repair capacity of cells [20]. Natural antioxidants in plants are categorized into three main groups: phenolic compounds, carotenoids, and vitamins. Phenolic compounds are structurally diverse, ranging from simple molecules (e.g., gallic, vanillic, ferulic, and caffeic acids) to polyphenols (e.g., flavonoids and tannins) [21]. For example, curcumin is a natural phenolic antioxidant extracted from rhizomes such as *turmeric* in the ginger family, and studies have shown that curcumin increases the expression of antioxidant enzymes against reactive oxygen species (ROS) by up-regulating AR expression through Nrf2 in a PI3K/Akt-dependent manner [22,23]. Mammalian Nrf2/CNC protein is recognized as an antioxidant regulator, and its immediate homologue in *C. elegans* is skn-1 [24]. *T. chinense* is rich in polyphenols, polysaccharides, alkaloids, volatile oils, amino acids, and other active ingredients, of which the main active ingredient AG is a typical polyphenolic compound. It is hypothesized that AG may also exert anti-aging and antioxidant effects through the skn-1 target, and a preliminary study of its hormesis dose effects has been conducted.

*Sirtuins*, which participate in NAD+-dependent deacetylation processes [25], are widely regarded as defensive anti-aging components due to the established connection between heightened sirtuin function and prolonged lifespan, as well as reduced function and the onset of age-related ailments [26]. Researchers produced a panel of isogenic human stem cell lines with *SIRT1*–*SIRT7* knockouts and found that any sirtuin deficiency leads to accelerated cellular senescence [27]. Resveratrol enhances the longevity of various model organisms through the modulation of oxidative stress, energy metabolism, nutrient perception, and epigenetic mechanisms [28]. This effect is predominantly achieved through the activation of sirtuin 1, a process that may be compromised in cases of NAD+ insufficiency [29].

Researchers have shown that *SIRT6* overexpression leads to a reduction in frailty and lifespan extension in both male and female B6 mice [30]. For example, celastrol and melatonin modify *SIRT1*, *SIRT6*, and *SIRT7* gene expression and improve the response of human granulosa-lutein cells to oxidative stress [31]. It is noteworthy that the overexpression of *SIRT6* increased IGF-1-binding proteins and altered the phosphorylation levels of IGF-1 signaling components, thereby inhibiting the IGF-1 pathway and prolonging the lifespan of model animals [32]. Senescent cells promote tissue NAD+ decline during aging via the activation of CD38 macrophages [33]. Reproductively young mice lacking CD38 exhibited larger primordial follicle pools, elevated ovarian NAD+ levels, and increased fecundity relative to wild-type controls. Mammalian female reproductive lifespan is typically significantly shorter than life expectancy and is associated with a decrease in ovarian NAD+ levels [34].

However, few studies have investigated the mechanism of action of AG in delaying aging and its role in fish culture. This research aimed to investigate the mechanism of action of AG in delaying aging and its role in fish culture. The study is generally classified into two parts: prediction and validation. Firstly, we predicted the interactions between AG and IGFR/CD38 using blind docking and molecular dynamics simulations. Then, we explored the lifespan extension effect of AG in *C. elegans* and the antioxidant effect of AG in the embryonic zebrafish, respectively, and verified the predicted targets by quantitative real-time PCR.

## 2. Materials and Methods

### 2.1. Chemicals and Reagents

LC-MS-grade acetonitrile (ACN) was purchased from Fisher Scientific (Fisher Scientific, Loughborough, Leicestershire, UK). Formic acid was obtained from Tokyo Chemical Industry (TCI-SCT Shanghai, China). Ammonium formate was obtained from Sigma-Aldrich (JT Baker, Phillipsburg, NY, USA). Ultrapure water was generated using a Milli-Q system (Millipore, Bedford, MA, USA). AG and DMSO were purchased from Aladdin (Aladdin, Shanghai, China). An ROS kit was bought from Nanjing Jiancheng Bioengineering Institute (Jiancheng, Nanjing, China). TRIzol Reagent and SuperScript^®^ II reverse transcriptase were obtained from Invitrogen (Invitrogen, Eugene, OR, USA). The quantitative real-time PCR (qRT-PCR) system was obtained from Takara Bio Inc. (Takara Bio Inc., Shiga, Japan). Thirty percent H_2_O_2_ was purchased from Nanjing Chemical Reagent Factory (NJ-Reagent, Nanjing, China). Other reagents and solvents were of analytical grade.

### 2.2. T. chinense Extract

*T. chinense* was cultivated on the campus of Nanjing Agricultural University (NJAU), harvested on 30 May 2023, and dried in an oven at 60 °C, No. BRC20230530, and stored at room temperature in the College of Life Sciences, Nanjing Agricultural University, B1022. The herbs harvested were identified by Prof. Zengxu Xiang of the College of Horticulture, Nanjing Agricultural University, as the dried whole herb of *T. chinense* in the *Sandalwood* family. *T. chinense* was soaked in ethanol (85%, 1:10 *w*/*v*) at room temperature for 24 h and then ultrasonically extracted at 60 °C for 2 h. The filtrate was combined, filtered, and evaporated with a rotary evaporator. The extract was named W. A portion of W (100 g) was suspended in 100 mL water and then extracted (1:1 *v*/*v*, 30 min) with ethyl acetate. The organic phases were evaporated and freeze-dried to obtain the extract named EA.

### 2.3. LC-MS/MS Analysis of Extract

LC-MS analysis was performed using a Vanquish UHPLC System (Thermo Fisher Scientific, Waltham, MA, USA) and Q Exctive focus (Thermo Fisher Scientific, Waltham, MA, USA) with ESI ion source. LC-MS method referred to Zelena and Want for specific conditions, placed in the Supplementary Materials [35,36].

### 2.4. Molecular Docking

The X-ray crystal structures of CD38 (PDB: 3DZK) and IGFR (PDB: 5FXS) were obtained from the Protein Data Bank. The protonation state of AG was set at pH = 7.4, and AG was expanded to a 3D structure using Open Babel [37]. AutoDock Tools (ADT3) were applied to prepare and parametrize the receptor protein and ligands. The docking grid documents were generated by AutoGrid of sitemap, and AutoDock Vina (1.2.0) was used for the docking simulation [38,39]. The optimal pose was selected to analysis interaction. Finally, the protein–ligand interaction figure was generated by PyMOL (2.5.0, Schrödinger, New York, NY, USA).

### 2.5. Molecular Docking Simulations

The molecular dynamics simulations were carried out with Desmond/Maestro noncommercial version 2022.1 as a molecular dynamic’s software [40]. TIP3P water molecules were added to the systems, which were then neutralized by 0.15 M NaCl solution. After minimization and relaxation of the system, the production simulation was performed for 100 ns in an isothermal-isobaric ensemble at 300 K and 1 bar. Trajectory coordinates were recorded every 100 ps. The molecular dynamics analysis was performed using Simulation Interaction Diagram from Desmond.

### 2.6. C. elegans Strains and Maintenance

Wild-type Bristol N2, transgenic nematode strains: CF1553 *sod-3* p:: GFP (muls84), and *Escherichia coli* OP50 (*E. coli* OP50) were obtained from Caenorhabditis Genetics Center (CGC). All nematodes were maintained on a nematode growth medium (NGM) plate with a layer of *E. coli* OP50 as food at 20 °C. To attain synchronization, *C. elegans* in the oviposition phase were relocated onto NGM plates devoid of *E. coli* OP50 bacteria for egg deposition overnight, following which they were subsequently extracted. L1-stage *C. elegans* hatched from the eggs were then transferred to new NGM plates until they reached the adult stage. AG was dissolved in DMSO and poured onto an NGM plate when the medium was 50 °C during the liquid stage (the final concentration of DMSO was less than 0.1%).

### 2.7. C. elegans Lifespan Assay

Synchronized L1 larvae were cultured on NGM containing 0, 50, 250, and 500 µg/mL of AG in *E. coli* OP50. *C. elegans* was transferred to the fresh NGM plates every day, and then all the alive, escaped, and dead data were recorded. *C. elegans* were scored as dead when they did not respond to repeated touching with a platinum wire.

### 2.8. ROS Level Assay in C. elegans

Synchronized L1 *C. elegans* were put on an NGM plate containing AG or not at 20 °C for 48 h. Then, the *C. elegans* were collected and washed 5 times to remove *E. coli* OP50. Stain ROS in *C. elegans* using the ROS kit according to the manufacturer’s instructions. The fluorescence intensity of ROS was read at an excitation wavelength of 488 nm and an emission wavelength of 525 nm. In addition, the *C. elegans* were placed on glass slides, and the levels of ROS were observed under a fluorescence microscope (OLYMPUS BX53, Beijing, China).

### 2.9. C. elegans Gene Expression Assay

The synchronized L4 larvae were incubated with or without AG at 20 °C, as described in the lifespan assay. After a 2-day incubation, adult *C. elegans* were collected for extracting total RNA according to the manufacturer’s protocol. Afterwards, cDNA was synthesized using reverse transcriptase (SuperScript^®^ II). Following this, real-time PCR was completed using the iQ ^TM^ SYBR ^R^ Green Supermix kit (Bio-Rad, Shanghai, China), and anti-proliferative factor expression was detected by the BIO-RADCFX48^TM^ real-time system (Bio-Rad, Shanghai, China). Lastly, the relative expression of the anti-proliferative genes was calculated based on the 2^_∆∆Ct^. Real-time PCR primer sequences can be found in the Supplementary Materials (Table S1).

### 2.10. Waterborne Exposure of Embryos from Zebrafish

Embryos from zebrafish (AB wild-type) were provided by YSY Biotech (Nanjing, China). After 7–9 h post-fertilization (hpf), embryos were pre-exposed to a medium containing AG (0, 20, 50, and 100 µg/mL) for 2 h. In order to prevent the reaction of AG with H_2_O_2_, the fish culture water containing AG was therefore removed after 2 h of treatment and replaced with fish culture water containing 0.22 mmol/L H_2_O_2_ to induce oxidative stress injury. The mounded embryos were incubated in a constant temperature incubator at 28 °C for 96 h. Embryonic mortality and malformation rates were counted.

### 2.11. Assay of ROS Level and Cell Death in Zebrafish Embryos 

Samples treated by method Section 2.10. were stained with DCFH-DA and acridine orange and incubated for the appropriate reaction time for each reagent. Embryos were then washed, anesthetized, and observed under a fluorescence microscope. Quantification of fluorescence staining intensity was performed and visualized by utilizing ImageJ 1.54 h software (NIH, Bethesda, MD, USA).

### 2.12. Zebrafish Embryos Gene Expression Assay

Zebrafish embryos were prepared as described in Section 2.10. The expression of antioxidant-related genes in zebrafish embryos was assayed according to Section 2.9. Real-time PCR primer sequences can be found in Supplementary Materials (Table S1).

### 2.13. Statistical Analyses 

Origin 2021 software was used for visualization of the data, presented as mean ± standard deviation (SD). The one-way analysis of variance (ANOVA) was performed using SPSS 20.0 software (IBM, Armonk, NY, USA) to analyze differences between groups. Different letters within the same group of treatments indicate a significant difference (*p* < 0.05). Graphical abstract was conducted by Figdraw (https://www.figdraw.com/, accessed on 15 July 2024).

## 3. Results

### 3.1. AG in EA of T. chinense 

**Figure 1:**
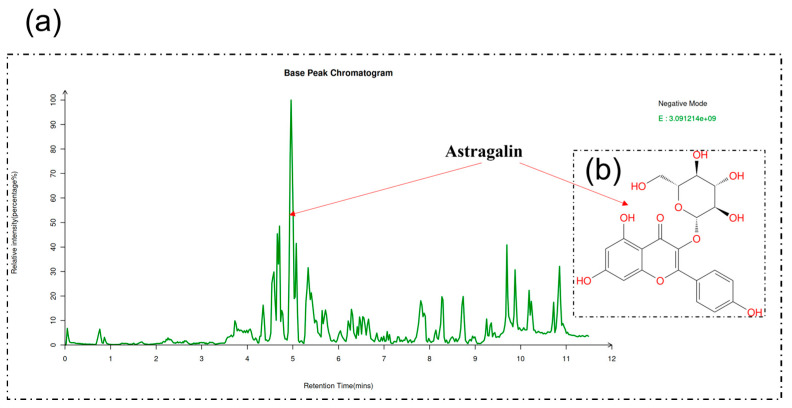
LC-MS/MS analysis of extract: (**a**) base peak chromatograms (BPCs) of EA of *T. chinense* and (**b**) chemical structures of AG.

AG in EA of *T. chinense* was analyzed by LC-MS with the following parameters: retention time: 4.9 min, MZ: 447.0935, exact mass: 448.1006, formula: C_21_H_20_O_11_, class: flavonoids, and CAS: 480-10-4 (Figure 1).

### 3.2. Molecular Docking of IGFR/CD38 with AG

The IGFR and CD38 proteins are represented as a slate cartoon model, the ligand is shown as a cyan stick, and their binding sites are shown as magenta stick structures. The hydrogen bond, ionic interactions, and hydrophobic interactions are depicted as yellow, magenta, and green dashed lines, respectively.

**Figure 2:**
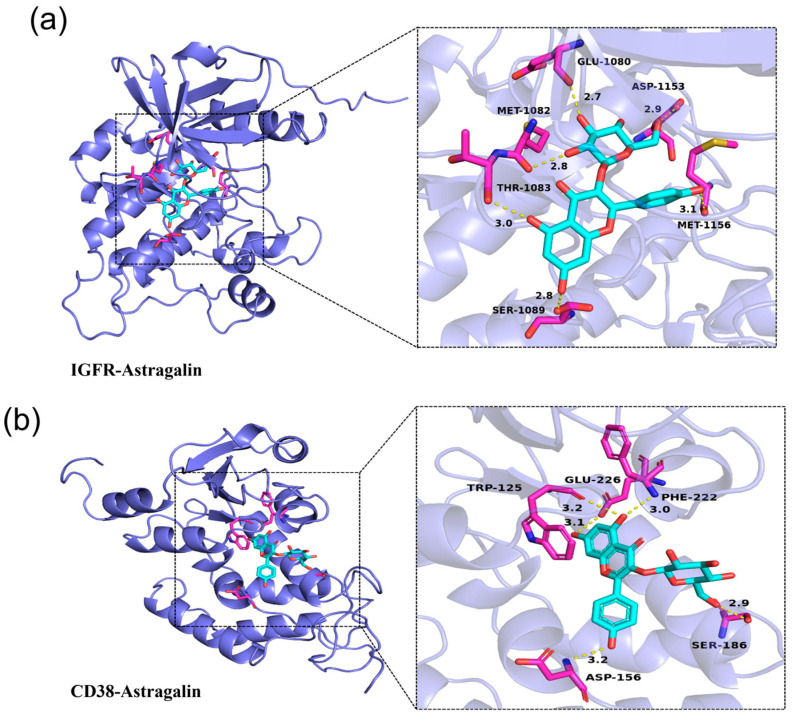
Molecular docking of IGFR/CD38 with AG: (**a**) docking of AG with IGFR; (**b**) docking of AG with CD38.

We analyzed the interactions between the protein and ligand, and all functional residues were identified and classified based on their interactions. There are multiple groups of residues used to form interactions between receptor protein and ligand, such as the hydrogen bond formed by (GLU1080, MET1082, THR1083, SER1089, ASP1153, MET1156) of IGFR-AG and (TRP125, ASP156, SER186, PHE222, GLU226) of CD38-AG (Figure 2). With these interaction forces, the binding energy of IGFR and CD38-AG complex was all −8.5 kcal/mol, which demonstrates excellent performance. However, the above results are all theoretical calculations and for reference only, everything is subject to experiment.

### 3.3. Molecular Dynamics Simulations of IGFR/CD38 with AG

**Figure 3:**
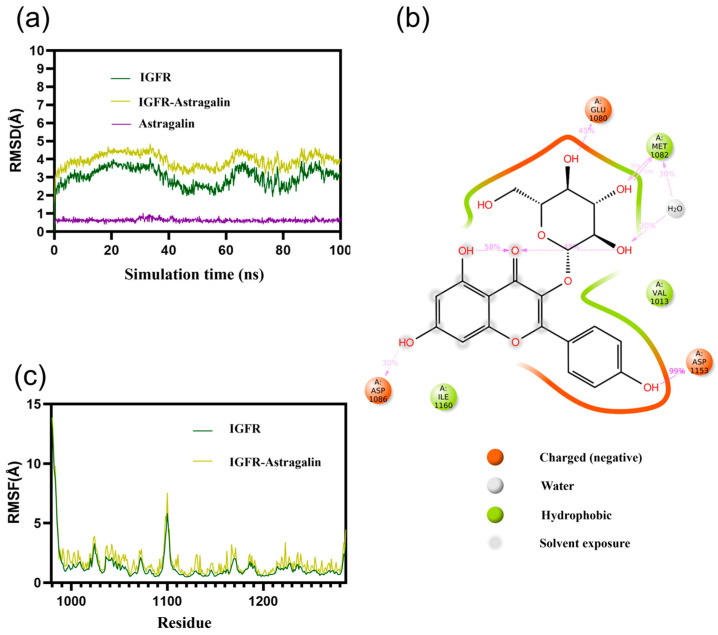
Molecular dynamics simulations of IGFR with AG: (**a**) RMSD of AG-IGFR; (**b**) two-dimensional protein–ligand interaction diagram between AG and IGFR; and (**c**) RMSF of AG-IGFR.

**Figure 4:**
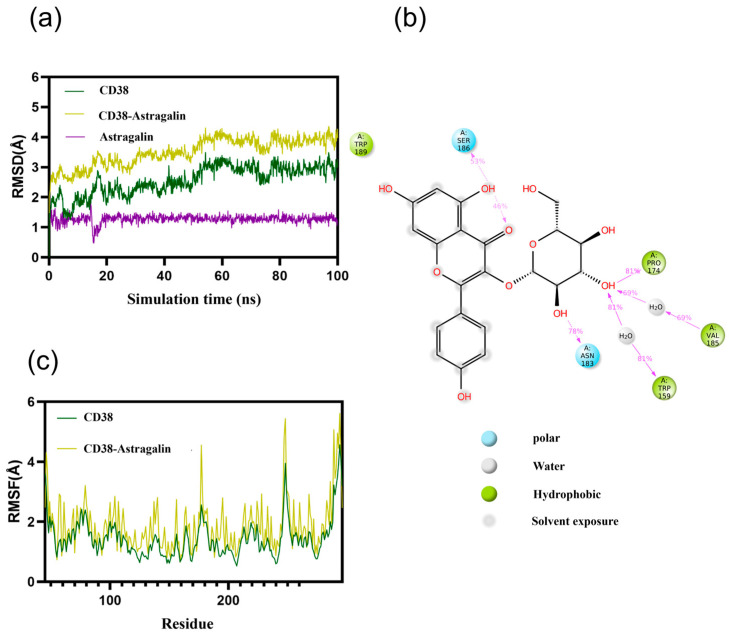
Molecular dynamics simulations of CD38 with AG: (**a**) RMSD of AG-CD38; (**b**) two-dimensional protein–ligand interaction diagram between AG and CD38; and (**c**) RMSF of AG-CD38.

The Root Mean Square Deviation (RMSD) value serves as an indicator of alterations in the stability of complex conformation. During the simulation, the RMSD of AG-IGFR and free IGFR has been in a relatively stable state and eventually stabilized between 3.0 and 4.5 Å. The RMSD of AG-CD38 and free CD38 has also been in a relatively stable state and finally stabilized between 2.5 and 4.5 Å. The results showed that AG binding to protein did not cause large fluctuations (Figure 3a and Figure 4a). Moreover, the RMSF value of flexibility of expressed amino acid residues indicated that complexes and free IGFR and CD38 amino acid fluctuations were relatively stable (Figure 3c and Figure 4c).

The main interactions of the IGFR along the trajectory were as follows: ionic interactions—GLU1080, ASP1153, ASP1086; hydrophobic interactions—MET1082, Val1013, Ile1160. It is worth mentioning that the hydrogen bond formation frequency of ASP1153 is 99%, suggesting that the ASP1153 amino acid plays a crucial role in the binding process. In addition, the ligand also formed two intramolecular hydrogen bonds for stabilizing the binding conformation of the small molecule (Figure 3b).

The main interactions of CD38 along the trajectory were as follows: hydrophobic interactions—TRP159, Val185, Pro174, TRP189; polar interactions—Asn183, SER186. It is noteworthy that the frequency of hydrogen bond formation for Pro174 is 81%, suggesting that the Pro174 amino acid plays a crucial role in the binding process. Additionally, the ligand also forms an intramolecular hydrogen bond to stabilize the binding conformation of the small molecule (Figure 4b) These results demonstrate the high stability of the IGFR and CD38 and AG complexes, and we could infer that AG could be depressants of the enzyme.

### 3.4. AG Extended the Lifespan of C. elegans 

**Figure 5:**
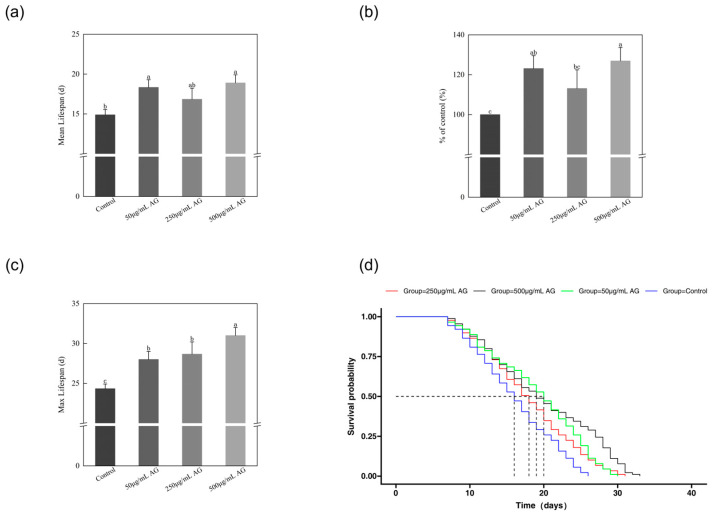
AG extended the lifespan of *C. elegans*: (**a**) effect of AG on the mean lifespan of N2 wild-type *C. elegans*; (**b**) effect of AG on the life extension rate of N2 wild-type *C. elegans*; (**c**) effect of AG on the survival curve of N2 wild-type *C. elegans*; (**c**) effect of AG on the max lifespan of N2 wild-type *C. elegans*; and (**d**) effect of AG on the survival curve of N2 wild-type *C. elegans*. The experiment was repeated at least three times. Notes: No common letter in the same treatment group indicates a significant difference (*p* < 0.05).

In *C. elegans*, inhibiting the insulin/*IGF-1* pathway through *daf-2*/*IGFR* inactivation proved to be a successful method of increasing lifespan. Our investigation focused on the longevity extension effect of caffeoylquinic acids in *C. elegans*, considering their high binding ability to the inhibitory regions of *IGFR*. The survival curves of all the nematodes were almost identical until day 11 (Figure 5c,d).

However, after day 12, the survival curves of the AG treatment groups shifted to the right relative to the control group, with the 500 µg/mL group showing the most noticeable shift. The average lifespan of the control nematodes was 15 days. After treatment with AG (50, 250, and 500 µg/mL), the mean longevity of the nematodes was recorded as 18, 17, and 19 days, respectively, demonstrating an increment of 23.13%, 13.13%, and 26.94% compared to the control cohort (Figure 5a,b).

### 3.5. AG Reduces ROS Levels of C. elegans 

**Figure 6:**
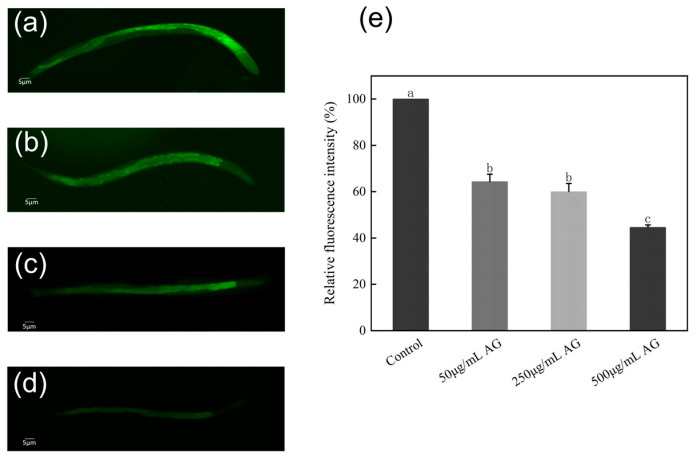
AG reduces ROS levels of *C. elegans*: (**a**) ROS levels of *C. elegans* treated without AG; (**b**) ROS levels of *C. elegans* treated with 50 µg/mL AG; (**c**) ROS levels of *C. elegans* treated with 250 µg/mL AG; (**d**) ROS levels of *C. elegans* treated with 500 µg/mL AG; and (**e**) effect of AG on the ROS of *C. elegans*. The experiment was repeated at least three times. In the absence of a common letter within the same treatment group, this indicates a significant difference (*p* < 0.05).

The control group had the highest levels of ROS, while treatment of AG (50, 250, and 500 µg/mL) decreased the relative ROS levels by 35.68%, 39.97%, and 55.38%, respectively. It is therefore hypothesized that AG may extend the lifespan of *C. elegans* by reducing the levels of ROS (Figure 6e).

### 3.6. AG Increases SOD Levels of C. elegans 

**Figure 7:**
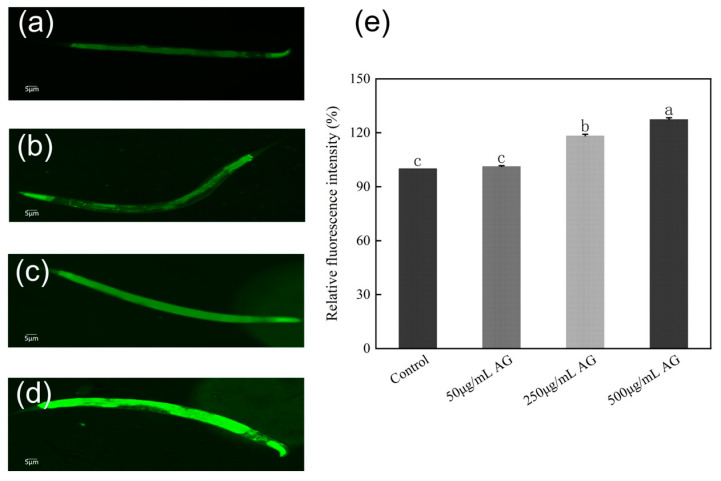
AG increases SOD levels of *C. elegans*: (**a**) *sod-3* p::GFP (muls84) treated without AG; (**b**) *sod-3* p::GFP (muls84) treated with 50 µg/mL AG; (**c**) *sod-3* p::GFP (muls84) treated with 250 µg/mL AG; (**d**) *sod-3* p::GFP (muls84) treated with 500 µg/mL AG; and (**e**) effect of AG on *sod-3* p::GFP (muls84). The experiment was repeated at least three times. Notes: No common letter in the same treatment group indicates a significant difference (*p* < 0.05).

The *C. elegans* treated with 500 µg/mL AG had the highest SOD levels, which were significantly higher than those of the control and other treatment groups (*p* < 0.05). However, 50 µg/mL AG had almost no effect on the levels of SOD in *C. elegans* (Figure 7). Therefore, we speculate that AG can prolong the lifespan by increasing the SOD activity in *C. elegans* when it reaches a certain dose.

### 3.7. AG Regulates the AMPK Pathway and Sirtuins-Associated mRNA Expression in C. elegans

**Figure 8:**
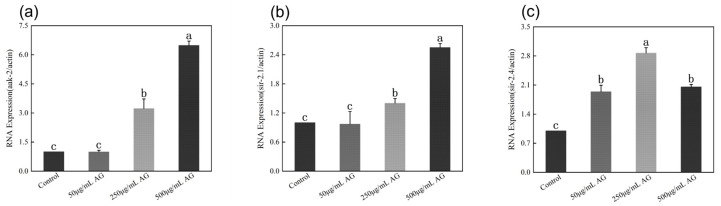
AG regulates the AMPK pathway and *Sirtuins*-associated mRNA expression in *C. elegans*: (**a**) effect of AG on *aak-2* expression in *C. elegans*; (**b**) effect of AG on *sir-2.1* expression in *C. elegans*; and (**c**) effect of AG on *sir-2.4* expression in *C. elegans*. The experiment was repeated at least three times. Note: No common letter in the same treatment group indicates a significant difference (*p* < 0.05).

*Sirtuins* are known to be protective anti-aging proteins because an increase in sirtuin activity is associated with increased longevity and a decrease in activity is linked to the development of aging-related diseases. Also, *Sirtuins* are mediators of calorie restriction and play an important role in ameliorating obesity and age-related metabolic diseases. The AMPK pathway is the upstream signaling that regulates the activity of *Sirtuins*, to determine whether AG has the potential to be a “Sirtfoods”, we chose homologous genes in *C. elegans* (*aak-2*, *sir-2.1*, and *sir-2.4*) and measured their expression. These results indicated that the expression of *aak-2*, *sir-2.1*, and *sir-2.4* was significantly increased when the concentration of AG was higher than or equal to 250 µg/mL, but there was no significant effect on the expression of *aak-2* and *sir-2.1* when the concentration of AG was 50 µg/mL (Figure 8).

### 3.8. AG Regulates the IIS Pathway and MAPK-Associated mRNA Expression in C. elegans

**Figure 9:**
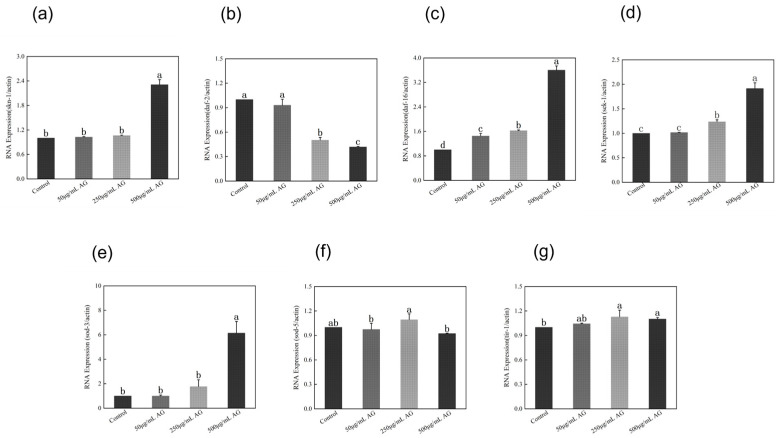
AG regulates the *IIS* pathway and AMPK-associated mRNA expression in *C. elegans*: (**a**) effect of AG on *skn-1* expressions in *C. elegans*; (**b**) effect of AG on *daf-2* expressions in *C. elegans*; (**c**) effect of AG on *daf-16* expressions in *C. elegans*; (**d**) effect of AG on *sek-1* expressions in *C. elegans*; (**e**) effect of AG on *sod-3* expressions in *C. elegans*; (**f**) effect of AG on *sod-5* expressions in *C. elegans*; and (**g**) effect of AG on *tir-1* expressions in *C. elegans*. The experiment was repeated at least three times. Note: No common letter in the same treatment group indicates a significant difference (*p* < 0.05).

Numerous studies have confirmed the mechanism of action by which *IIS* regulates *daf-16* to extend lifespan. To investigate that AG mediates lifespan in an *IIS* pathway-dependent manner, we assessed mRNA expression of transcription factors associated with the *IIS* pathway. AG down-regulated the expression of *daf-2* (*p* < 0.05), which was consistent with the results of the preliminary molecular docking and molecular dynamics simulations, proving that AG could bind to *IGFR*/*daf-2* intensively and exert an inhibitory effect (Figure 9).

In addition, 500 µg/mL of AG significantly up-regulated the expression of *daf-16*, *skn-1*, and *sod-3* (*p* < 0.05), which is consistent with the results of the SOD levels assay. However, AG had almost no effect on *sod-5* expression. Notably, the regulation of *skn-1* is usually dependent on the MAPK pathway. The examination was conducted on the expression of two crucial transcription factors, *sek-1* and *tir-1*, within the MAPK pathway (Figure 9). The results revealed a significant increase in the expression levels of both factors when the concentration of AG reached or exceeded 250 µg/mL (*p* < 0.05).

### 3.9. AG Inhibits H_2_O_2_-Induced Malformation Rate and Cell Death in Zebrafish Embryos

**Figure 10:**
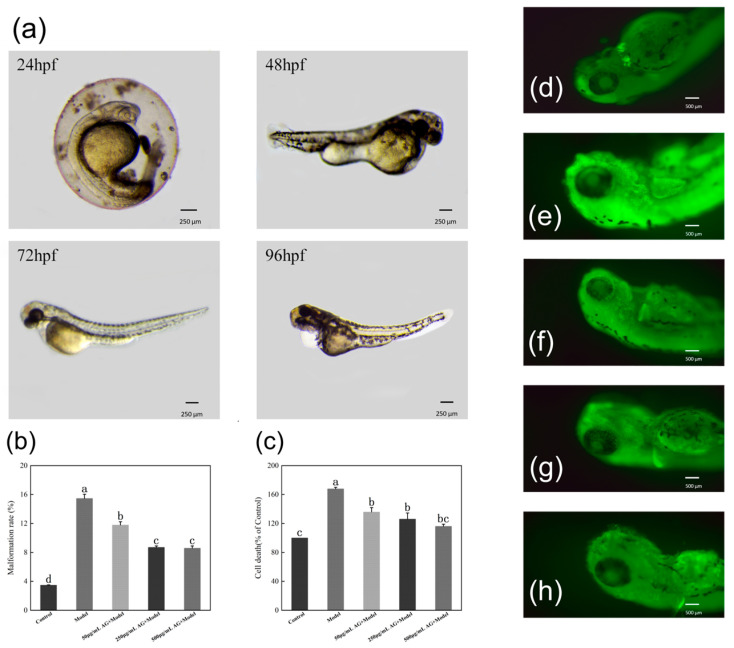
AG inhibits H_2_O_2_-induced malformation rate and cell death in zebrafish embryos: (**a**) H_2_O_2_ -induced damage in zebrafish embryos; (**b**) effect of AG on the malformation rate of zebrafish embryos; (**c**) effect of AG on the rate of cellular mortality in zebrafish embryos; (**d**) cell death of zebrafish embryos without AG and H_2_O_2_; (**e**) cell death of zebrafish embryos with H_2_O_2_; (**f**) cell death of zebrafish embryos with 50 µg/mL AG; (**g**) cell death of zebrafish embryos with 250 µg/mL AG; and (**h**) cell death of zebrafish embryos with 500 µg/mL AG. The experiment was repeated at least three times. Note: No common letter in the same treatment group indicates a significant difference (*p* < 0.05).

Oxidative stress leads to malformations such as damage to the egg membrane, abnormal curvature of the spine, and edema of the pericardium in zebrafish embryos. However, after AG treatment, AG (50, 250, and 500 µg/mL) significantly reduced the malformation rate of zebrafish embryos relative to the model group (*p* < 0.05) (Figure 10a,b). In addition, the rate of cellular mortality in zebrafish embryos exhibited a notable decrease subsequent to the administration of AG in comparison to the control group (*p* < 0.05) (Figure 10c–h).

### 3.10. AG Reduces ROS Levels of Zebrafish Embryos

**Figure 11:**
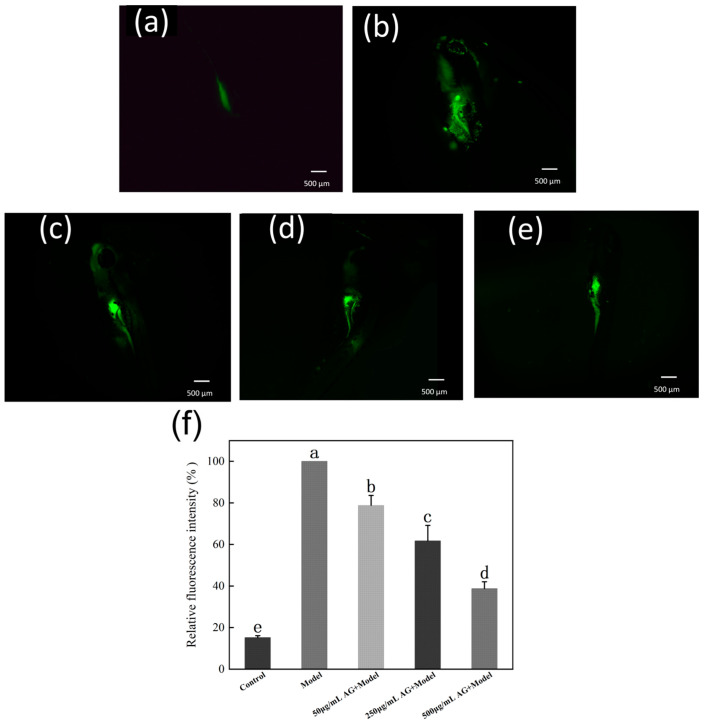
AG reduces ROS levels of zebrafish embryos: (**a**) ROS of zebrafish embryos without AG and H_2_O_2_; (**b**) ROS of zebrafish embryos with H_2_O_2_; (**c**) ROS of zebrafish embryos with 50 µg/mL AG; (**d**) ROS of zebrafish embryos with 250 µg/mL AG; (**e**) ROS of zebrafish embryos with 500 µg/mL AG; and (**f**) effect of AG on the ROS of zebrafish embryos. The experiment was repeated at least three times. Note: No common letter in the same treatment group indicates a significant difference (*p* < 0.05).

Similar to the trend of AG on H_2_O_2_-induced malformation rate in zebrafish embryos, after treatment with AG, the level of ROS within zebrafish embryos was significantly reduced compared to the model group (*p* < 0.05) (Figure 11). It is hypothesized that AG may exert antioxidant effects by reducing ROS levels in oxidatively damaged zebrafish embryos.

### 3.11. AG Regulates Sirtuins-Associated mRNA Expression in Zebrafish Embryos

**Figure 12:**
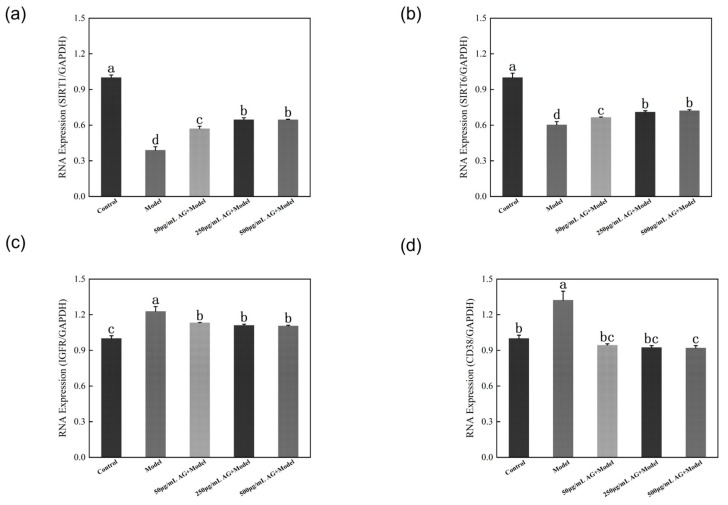
AG regulates *Sirtuins*-associated mRNA expression in zebrafish embryos: (**a**) effect of AG on *SIRT1* expressions in zebrafish embryos; (**b**) effect of AG on *SIRT6* expressions in zebrafish embryos; (**c**) effect of AG on *IGFR* expressions in zebrafish embryos; and (**d**) effect of AG on *CD38* expressions in zebrafish embryos. The experiment was repeated at least three times. Note: No common letter in the same treatment group indicates a significant difference (*p* < 0.05).

CD38 is an NAD+-depleting enzyme that regulates the expression of *Sirtuins* by affecting changes in NAD+ levels. Since there is no homologous gene for *CD38* in *C. elegans* but there is in zebrafish embryos, we used zebrafish embryos as a model animal to investigate the role of AG in the regulation of *CD38* during oxidative stress (Figure 12a). The outcomes of molecular docking and molecular dynamics simulations revealed a significant binding energy demonstrated by AG towards *CD38* and *IGFR*. This observation aligns with the notable down-regulation of both *CD38* and *IGFR* expressions in the treated cohort compared to the oxidative damage cohort post AG intervention, as evidenced by statistical significance (*p* < 0.05). Interestingly, after AG treatment, two important members of the *Sirtuins* gene family (*SIRT1* and *SIRT6*) were significantly up-regulated compared to the oxidative damage group (*p* < 0.05) (Figure 12b–d).

## 4. Discussion

Increased oxidative stress has been associated with the aging process [41]. Oxidative injury could potentially exacerbate the oxidative load associated with typical aerobic cellular processes, a phenomenon that inherently produces oxidizing agents, leads to the accumulation of oxidative damage within mitochondria, and participates in the natural aging process. The increase in oxidative damage may, in part, contribute to stress-associated acceleration of aging [42]. Many types of natural compounds, such as flavonoids and vitamins, have been shown to exert antioxidant and anti-aging effects in vivo and in vitro [43]. For example, the cellular levels of ROS and antioxidant enzymes were regulated by the main flavonoids of propolis extract, demonstrating both beneficial antioxidant and pro-oxidant effects [44]. Inspired by the effects of reversing oxidative stress through pretreatment with tomato and rosemary extracts [45], we found that AG had a similar effect. AG is a common flavonoid derived from *T. chinense*, which is also found in a wide range of edible plants (*Paeonia lactiflora* [46], *Artemisia absinthium L*. [47], and *Rhodomyrtus tomentosa* [48]). *T. chinense* has been receiving increasing attention for its applications and research recently. Therefore, in addition to using molecular docking and molecular dynamics simulation techniques, we also innovatively conducted a study on the anti-aging and antioxidant activities of AG using *C. elegans* and zebrafish embryos. 

ROS plays a role in the aging process of cells and contributes to various physiological signaling pathways [49]. According to the free radical theory of aging, aging is a result of the accumulated damage caused by ROS [50]. Oxygen-free radicals are believed to be involved in the aging process. Superoxide dismutase (SOD) is crucial for antioxidative defense [51] and is one of the most effective mechanisms in physiology for neutralizing reactive oxygen species [52]. The current study revealed that the lifespan and SOD levels were enhanced, and ROS levels were reduced, in *C. elegans* treated with 500 µg/mL AG.

Molecular docking and molecular dynamics simulation techniques are commonly used to uncover drug–protein interactions [53] and elucidate the mechanism of drug action [54]. For instance, two natural depressants of xanthine oxidase were identified through molecular docking and molecular dynamics simulations [55]. In this study, molecular docking and molecular dynamics simulations were employed to investigate AG, which binds to IGFR and CD38. IGFR and CD38 are key proteins of oxidative stress. The binding energies of the complexes were all −8.5 kcal/mol, and the RMSD and RMSF values of the complexes remained relatively stable. These results indicate that AG binds securely to IGFR and CD38 proteins during MD simulation. The results were validated through experiments on *C. elegans* and zebrafish embryos. Consequently, it is suggested that AG may exhibit antioxidant and anti-aging effects by targeting IGFR and modulating CD38 activity.

A study found the insulin/IGF receptor homolog *daf-2* regulates aging in *C. elegans*. Decreasing *daf-2* activity causes fertile adults to remain active much longer than normal and to live more than twice as long [56]. This could be because reduced *daf-2* signaling leads to changes in downstream targets via the *daf-16* gene, a fork-head transcription factor, which is regulated by *daf-2*, resulting in an extended lifespan [57]. Another study discovered that *Acrolein* promotes aging and oxidative stress via the stress response factor *daf-16*/*FOXO* in *C. elegans* [58]. In the present study, we observed a reduction in the expression of daf-2 and the expression of daf-16 in *C. elegans* treated with 500 µg/mL AG. Therefore, we hypothesized that AG might have an anti-aging effect by inhibiting the activity of daf-2/IGFR through binding to daf-2/IGFR, thereby activating daf-16.

Decreased NAD+ levels have been shown to contribute to metabolic dysfunction during aging. NAD+ decline can be partially prevented by knockout of the enzyme CD38 [59]. In *CD38* knockout mice, tissue levels of NAD+ are significantly increased [60]. In d-gal-induced acute aging mice, *CD38* and *SIRT6* exhibited increased and decreased expression, respectively, in myocardial tissues. This is because *CD38* down-regulates *SIRT6* expression to promote cell senescence [61]. *SIRT6*, a nuclear histone deacetylase, functions at the chromatin level to directly attenuate IGF signaling. *SIRT6*-deficient mouse hearts exhibited hyperactivation of IGF signaling-related genes and their downstream targets. Mechanistically, *SIRT6* binds to and suppresses the promoter of IGF signaling-related genes by interacting with c-Jun and deacetylating histone 3 at Lys9 (H3K9) [62]. Similar to dietary restriction, mice overexpressing the NAD+-dependent protein deacylase *SIRT6* (MOSES) live longer and have reduced *IGF-1* levels [63].

AMPK activates histone deacetylases (HDACs) and *Sirtuins* by increasing the cellular concentration of NAD+, a cofactor of *Sirtuins* [64]. The AMPK signaling pathway regulates a comprehensive signaling network that plays a role in governing both healthspan and lifespan, for instance, through the modulation of *SIRT1* signaling cascades [65]. Moreover, caloric restriction is believed to slow down aging by boosting the activity of some *Sirtuins* through activating adenosine monophosphate-activated protein kinase (AMPK), thus raising the level of intracellular nicotinamide adenine dinucleotide NAD+ by stimulating NAD+ biosynthesis [66]. In the present study, we found that the expression of aak-2, sir-2.1, and *sir-2.4* was increased in *C. elegans* treated with 500 µg/mL AG. Therefore, we hypothesized that AG may play an anti-aging role by activating the AMPK pathway to increase the level of NAD+, consequently activating the expression of the *Sirtuins* genes.

In *C. elegans*, the *skn-1* gene encodes a transcription factor that resembles mammalian Nrf2 and activates a detoxification response. *Skn-1* promotes resistance to oxidative stress (Oxr) and also increases lifespan. It has been suggested that the former causes the latter, consistent with the theory that oxidative damage causes aging [67]. Furthermore, the *skn-1* transcription factor is considered by some to be an evolutionarily conserved regulator of exogenous stress and longevity [68]. Importantly, *skn-1* plays a central role in diverse genetic and pharmacologic interventions that promote *C. elegans* longevity, suggesting that mechanisms regulated by skn-1 may be of conserved importance in aging. These *C. elegans* studies predict that mammalian Nrf/CNC protein functions and regulation may be similarly complex and that the proteins and processes that they regulate are likely to have a major influence on mammalian life and healthspan [24]. *Skn-1* is mainly regulated by the upstream signaling pathway MAPK [69]. In this study, we found that the expressions of *sek-1*, *tir-1*, *skn-2*, and *sod-3* were all elevated in *C. elegans* treated with 500 µg/mL AG; so, we hypothesized that AG may play an anti-aging role by activating *sek-1* and *tir-1* in the MAPK pathway, which increases the expression of *skn-1* and thus activates its downstream signal, *sod-3*.

**Figure 13:**
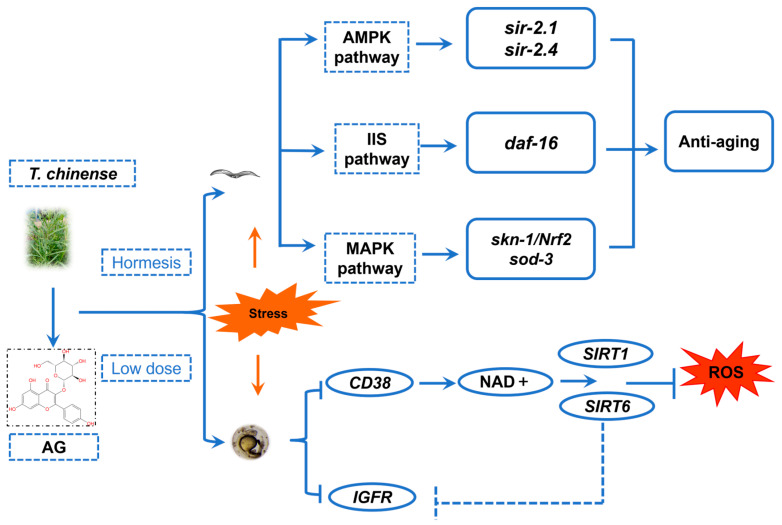
The schematic mechanism of action of AG as hormesis nutrition in anti-aging and antioxidant process: The figure represents hormesis and nutrients (AG) and the involvement of *skn-1/Nrf2*, *daf-16*, *sod-3*, *SIRT1*, and *SIRT6* genes to enhance antioxidant activity and stresses resistance and the inhibition of *IGFR/daf-2* and *CD38* genes via the activation of AMPK/ MAPK/IIS pathway in order to regulate the lifespan of *C. elegans* and the antioxidant capacity of zebrafish embryos by inducing anti-aging and antioxidant effects dose dependently [70].

Similarly, in zebrafish embryos, after AG treatment, the levels of *SIRT1* and *SIRT6* in oxidatively damaged zebrafish embryos were increased and the levels of ROS, *IGFR*, and *CD38* were reduced. Combining the results of molecular docking and molecular dynamics simulation, we speculate that AG can not only directly bind to *IGFR* to inhibit its activity but also bind to *CD38* to inhibit the activity of *CD38*, increase the level of NAD+, activate the expression of *SIRT1* and *SIRT6*, and inhibit the activity of *IGFR* by up-regulation of *SIRT6*, thus synergistically exerting the antioxidant effect (Figure 13).

## 5. Conclusions

This study demonstrated that a typical flavonoid from *T. chinense*, AG, could increase the expression of *daf-16* by inhibiting the expression of *IGFR/daf-2*, thereby prolonging the lifespan of *C. elegans*. It is worth mentioning that after the preventive treatment with AG, both *IGFR* and *CD38* were inhibited in oxidatively damaged zebrafish embryos, and ROS content was significantly decreased, which improved the resistance to oxidative stress in zebrafish embryos. In addition, in *C. elegans*, AG activated transcription factors related to the AMPK/ MAPK/IIS pathway despite the inhibition of *IGFR/daf-2*, thereby regulating the lifespan of *C. elegans*. However, the interactions among transcription factors need further in-depth study. In summary, the previous studies further demonstrated the safety of AG and its anti-aging and antioxidant efficacy. These results help to explain the anti-aging effects of AG on *C. elegans*. The results also support the use of AG as a feed additive to enhance the antioxidant capacity of aquatic products.

However, there are some limitations of this study. For instance, *C. elegans* are deficient in several mammalian metabolic pathways, such as cardiovascular and hepatic first-pass metabolism. Furthermore, we did not utilize *C. elegans* mutant (e.g., EU1 and CL2166, LG348, LG357) knockout mice to further validate the antioxidant and anti-aging mechanisms of AG; therefore, potential directions for our future research may include the application of animal models (e.g., mice, monkeys) as well as the *C. elegans* mutant or implementing clinical trials to explore the antioxidant and anti-aging properties of AG. 

## Data Availability

The data presented in this study are available in the article.

## Supplementary Materials

The following supporting information can be downloaded at: https://www.mdpi.com/article/10.3390/antiox13070859/s1, Table S1. List of used for rt-PCR assays.
