# Supplementary material for: Astragalin from Thesium chinense: A Novel Anti-Aging and Antioxidant Agent Targeting IGFR/CD38/Sirtuins
Source: Antioxidants (Basel). 2024 Jul 18;13(7):859. doi: 10.3390/antiox13070859 (PMC11273813; doi:10.3390/antiox13070859)
Supplement: Supplementary file 1 [file antioxidants-13-00859-s001.zip › antioxidants-3089542-supplementary.pdf]

Table S1 List of used for rt-PCR assays

Gene's name	Forward (5'-3')	Reverse (5'-3')
daf-16	ACATTGCTCGAAGTGCCGAA	CATTGCTGTCGACCCGTTTG
daf-2	GCTTACGCGATGAGCTGTGAT	TCGCTGGCGACTATGTGA
aak-2	TCTTCCGCCATCCGCATATC	CCTCTTCATCGGGTCTACGC
sir-2.4	GGTGTGAGCACTGGATCGAA	GGACACAGGCCAACACTCAT
sod-3	CAATTGCTCTCCAACCAGCG	TTTGCACAGGTGGCGATCTT
pmk-1	TCCGACTCCACGAGAAGGAT	TCCGACTCCACGAGAAGGAT
tir-1	AAAGAGTTTGAGCCACTG	CTGCTCCTTCTTAATAGTTG
nsy-1	CTGTTCTCCGTCGCCTTGAT	GGTGGAGTACGGAATGGTGG
sek-1	AGCCGGATGCAAACCTTACA	CATCGTCGCCAAACAGTGTC
sir-2.1	GTTCGTGTGGTTCGTCTTGC	CGCGTTTTTCAATGGGATTTGG
skn-1	ACTCTTTCCTCCCTTCGGAT	GGTGTATGGGGGTGAACAGG
Sirt1	TTCAGTGCCACGGGTCTTTT	GGACACCTGGGACAATGAGG
Sirt6	TGGTGTGTGGACGATGGAAG	CTCCGACAATCGATCCCTGG
igf1ra	GATCCAAAGAGCAGGGCTCC	CCCTCGTCATGCCAAAGTCT
CD38	AGCAGAATTTGGGACCACCC	TCGAACTCCTGCCATACAGC

Liquid chromatography conditions

The LC analysis was performed on a Vanquish UHPLC System (Thermo Fisher Scientific, USA). Chromatography was carried out with an ACQUITY UPLC ® HSS T3 (2.1×100 mm, 1.8 µm) (Waters, Milford, MA, USA). The column maintained at 40 ℃. The flow rate and injection volume were set at 0.3 mL/min and 2 µL, respectively. For LC-ESI (+)-MS analysis, the mobile phases consisted of (B2) 0.1% formic acid in acetonitrile (v/v) and (A2) 0.1% formic acid in water (v/v). Separation was conducted under the following gradient: 0~1 min，8% B2；1~8 min，8%~98% B2；8~10 min，98% B2；10~10.1 min，98%~8% B2；10.1~12 min，8% B2. For LC-ESI (-)-MS analysis, the analytes was carried out with (B3) acetonitrile and (A3) ammonium formate (5mM). Separation was conducted under the following gradient: 0~1 min，8% B3；1~8 min，8%~98% B3；8~10 min，98% B3；10~10.1 min，98%~8% B3；10.1~12 min，8% B3 [1].

Mass spectrum conditions

Mass spectrometric detection of metabolites was performed on Q Exactive Focus (Thermo Fisher Scientific, USA) with ESI ion source. Simultaneous MS1 and MS/MS (Full MS-ddMS2 mode, data-dependent MS/MS) acquisition was used. The parameters were as follows: sheath gas pressure, 40 arb; aux gas flow, 10 arb; spray voltage, 3.50 kV and -2.50 kV for ESI(+) and ESI(-), respectively; capillary temperature, 325 ℃; MS1 range, m/z 100-1000; MS1 resolving power, 70000 FWHM; number of data dependant scans per cycle, 3; MS/MS resolving power, 17500 FWHM; normalized collision energy, 30 eV; dynamic exclusion time,utomatic[2].

Reference

[1] Zelena E, Dunn W B, Broadhurst D, et al. Development of a Robust and Repeatable UPLC-MS Method for the Long-Term Metabolomic Study of Human Serum[J]. Analytical Chemistry, 2009,

81(4): 1357-1364.
[2] Want E J, Masson P, Michopoulos F, et al. Global metabolic profiling of animal and human tissues via UPLC-MS[J]. Nature Protocols, 2013, 8(1): 17-32.
